# Metabolite profiling in identifying metabolic biomarkers in older people with late-onset type 2 diabetes mellitus

**DOI:** 10.1038/s41598-017-01735-y

**Published:** 2017-06-29

**Authors:** Zhi Yang Tam, Sean Pin Ng, Ling Qiao Tan, Chih-Hsien Lin, Dietrich Rothenbacher, Jochen Klenk, Bernhard Otto Boehm, SPC Team, Kelvin Goh Kau Kiat, Pipob Suwanchaikasem, Pornpimol Tipthara, Song Yi Yang, ActiFE Study Group, T. Becker, J. Stingl, W. Koenig, M. Riepe, R. Peter, H. Geiger, A. Ludolph, C. v. Arnim, G. Nagel, G. Weinmayr, K. Rapp, M. D. Denkinger, D. Dallmeier, J. M. Steinacker, R. Laszlo

**Affiliations:** 1 Singapore Phenome Center, Experimental Medicine Building, Nanyang Technological University, 59 Nanyang Drive, Singapore, 636921 Singapore; 2 Lee Kong Chian School of Medicine, Nanyang Technological University, 59 Nanyang Drive, Singapore, 636921 Singapore; 3 Institute of Epidemiology and Medical Biometry, Ulm University, Helmholtzstrasse 22, 89081 Ulm, Germany; 4 Department of Clinical Gerontology, Robert-Bosch-Hospital, Auerbachstrasse 110, 70376 Stuttgart, Germany; 5 Division of Diabetes, Endocrinology and Metabolism, Department of Medicine, Imperial College London, London, UK; 6 Department of Internal Medicine I, Ulm University Medical Centre, Ulm University, Albert-Einstein-Allee 23, 89081 Ulm, Germany; 7 Department of Psychiatry and Psychotherapy II, Ulm University, Ulm, Germany; 8 Department of Dermatology and Allergic Diseases, Ulm University, Ulm, Germany; 9 Institute of Pharmacology of Natural Products & Clinical Pharmacology and BfARM (Federal Institute for Drugs and Medical Devices), Bonn, Germany; 10 Department of Internal Medicine II-Cardiology, University of Ulm Medical Center and German Heart Centre, Technical University Munich, Munich, Germany; 11 DZHK (German Centre for Cardiovascular Research) Munich, Munich, Germany; 12 Division of Gerontopsychiatry, Department of Psychiatry and Psychotherapy II, Ulm University, Ulm, Germany; 13 Institute of the History, Philosophy and Ethics of Medicine, Ulm University, Ulm, Germany; 14 Department of Molecular Medicine, Ulm University, Ulm, Germany; 15 Department of Neurology, Ulm University, Ulm, Germany; 16 Institute of Epidemiology and Medical Biometry, Ulm University, Ulm, Germany; 17 Department of Geriatrics and Geriatric Rehabilitation, Robert-Bosch-Hospital Stuttgart, Stuttgart, Germany; 18 Agaplesion Bethesda Clinic, Ulm, Germany; 19 Division of Sports and Rehabilitation, Department of Internal Medicine II–Cardiology, Ulm University Medical Center, Ulm, Germany

## Abstract

Regulation of blood glucose requires precise coordination between different endocrine systems and multiple organs. Type 2 diabetes mellitus (T2D) arises from a dysregulated response to elevated glucose levels in the circulation. Globally, the prevalence of T2D has increased dramatically in all age groups. T2D in older adults is associated with higher mortality and reduced functional status, leading to higher rate of institutionalization. Despite the potential healthcare challenges associated with the presence of T2D in the elderly, the pathogenesis and phenotype of late-onset T2D is not well studied. Here we applied untargeted metabolite profiling of urine samples from people with and without late-onset T2D using ultra-performance liquid-chromatography mass-spectrometry (UPLC-MS) to identify urinary biomarkers for late-onset T2D in the elderly. Statistical modeling of measurements and thorough validation of structural assignment using liquid chromatography tandem mass-spectrometry (LC-MS/MS) have led to the identification of metabolite biomarkers associated with late-onset T2D. Lower levels of phenylalanine, acetylhistidine, and cyclic adenosine monophosphate (cAMP) were found in urine samples of T2D subjects validated with commercial standards. Elevated levels of 5′-methylthioadenosine (MTA), which previously has only been implicated in animal model of diabetes, was found in urine of older people with T2D.

## Introduction

Diabetes mellitus is a highly prevalent chronic disorder characterized by complex changes of metabolism in the body systems. In type 2 diabetes mellitus (T2D), the inability of the body to maintain glucose homeostasis stems from a combination of insulin resistance and beta-cell dysfunction^1^. If unrecognized or inadequately treated, T2D can cause organ damage in every system and lead to cardiovascular diseases (CVD), nephropathy, retinopathy, and neuropathy^2^. Globally, there is a dramatic increase in the incidence and prevalence of T2D in all age groups, particularly in older people. In 2012, 4.8 million deaths were caused by T2D alone, most of them by CVD^3^. The prevalence of diabetes in the world is 8.3% in 2013, and is projected to increase further in the future^4^. By the year of 2035, an estimated of 592 million individuals will be affected by this disease, as compared to 382 million in the year of 2013^4^. This in turn will lead to a significant increase in healthcare expenditure worldwide^5^. The prevalence of diabetes is highest amongst the oldest age groups, and diabetes in older people is linked to higher mortality, reduced functional status, and higher risk of institutionalization^6^. It is therefore a major health issue that will most certainly grow in magnitude as the population of modern societies continues to age^7^.

Despite the increasing prevalence and greater costs associated with diabetes in the older population, the epidemiology and pathogenesis of late-onset T2D is still not well understood^8^. Furthermore, older people are often excluded from T2D related interventional trials^8^. The current panel of more than 80 T2D associated genetic variants are known to refer to patients with a disease-onset before 60 years of age^9, 10^. Although the decline in glucose tolerance as part of human aging is well established, not much is known about the metabolic profile in people with late-onset T2D^11–13^.

Metabolomics study involves the use of high-throughput technologies, e.g. liquid chromatography tandem mass spectrometry (LC-MS) and nuclear magnetic resonance (NMR), to comprehensively identify and quantify all or selected groups of endogenous small molecule metabolites^14^. This new approach has been successfully applied to describe metabolic phenotypes in T2D^15^. Using this methodology, the metabolisms of amino acid, carbohydrates, and lipids, in pathways such as glycolysis, gluconeogenesis, tricarboxylic acid cycle, lipolysis, and proteolysis, were found to be significantly altered in different stages of T2D^16–18^. It is important to note that the subjects included in these previous studies^16, 19^, were often less than 70 years old, and the age of disease onset was also before 70 years of age. As a result, the metabolic profile of older adults with late-onset T2D is still not well characterized.

In the light of getting a better insight into the complex age-associated changes in fuel metabolism and regulation related to diabetes, we have studied a cohort of community-dwelling older people from the Southern part of Germany. In this case-control study, untargeted urine metabolomics was performed on a cohort of older people aged 70 years and older with late-onset type 2 diabetes mellitus and respective controls^20^. In addition, data obtained were used to compare with the already published results from metabolomics studies in T2D subjects where the disease onset has been before 70 years of age.

## Results

### Baseline characteristics

The nested case and control cohort (see Table 1) consists of 80 older people with late-onset type 2 diabetes mellitus and 79 older controls without T2D. Presence of T2D was defined according to ADA 2010 criteria (Diagnosis and Classification of Diabetes Mellitus)^21^. For older people with diabetes, the median age of disease manifestation was 73 years. In the diabetes group, 35% of the participants were female, compared to 50% in the control group. The median age for older people with diabetes and control subjects was 81.3 years and 73.8 years, respectively. Older people with T2D were significantly older than control subjects (*p* < 0.001) and had higher levels of serum creatinine, cystatin C, and BMI. In comparison to subjects without diabetes, older people with diabetes were also found to have lower levels of eGFR, cholesterol, and serum calcium. In the T2D group, 48.8% had statin medication while 38.8% were treated with metformin. In the control group, 29.5% were on statins, while none were treated with metformin^22^. Using linear regression, metformin was not found to be associated with cholesterol level (β = 0.04, p = 0.89). Statin use was found to be significantly correlated with cholesterol level (β = −1.02, p < 0.001) in a model that adjusted for sex (β = 0.60, p < 0.001) and age (β = −0.02, p = 0.08). Cholesterol levels were found to be significantly different (p < 0.001) in control and T2D group using a bivariate (crude) comparison (Mann Whitney U test). However, after adjusting for age, gender, and statin medication, the difference in cholesterol level between control and T2D group was no longer significant (β = 0.26, p = 0.15).

**Table 1:** Baseline characteristic of a nested case and control participants from ActiFE study (n = 159).

	T2D (N = 80)	Control (N = 78)	*p* value**
Female (%)	36	51	
Metformin Medicated (%)	38.8	0	<0.001
Statins Medicated (%)	48.8	29.5	0.015
Age	81.25 (78.23–83.55)	73.80 (69.80–82.05)	<0.001
BMI	28.80 (26.02–31.30)	26.60 (24.80–28.70)	0.006
Glucose (mg/dL)	122.00 (103.75–145.00)	99.00 (93.00–108.00)	<0.001
Creatinine (μmol/l)	98.5 (82.00–118.25)	79.50 (73.00–93.00)	<0.001
eGFR-SCr & SCys (ml/min/1.73 m^2^)	65.2 (50.44–75.34)	81.55 (68.16–91.72)	<0.001
eGFR-SCr (ml/min/1.73 m^2^)	56.44 (46.25–68.92)	70.23 (58.87–82.80)	<0.001
eGFR-SCys (ml/min/1.73 m^2^)	67.02 (55.20–82.15)	89.34 (70.52–101.08)	<0.001
Cystatin C (mg/l)	1.02 (0.88–1.16)	0.83 (0.74–0.99)	<0.001
Urea (mmol/l)	6.90 (5.10–8.45)	5.90 (5.10–7.05)	0.056
Uric acid (μmol/l)	349.00 (292.50–416.25)	309.50 (270.25–374.75)	0.049
Cholesterol (mmol/l)	4.90 (4.10–5.63)	5.60 (4.83–6.28)	<0.001
LDL cholesterol (mmol/l)	3.20 (2.48–3.53)	3.70 (2.93–4.18)	0.001
HDL cholesterol (mmol/l)	1.30 (1.10–1.50)	1.40 (1.20–1.70)	0.004
GGT (U/l)	24.00 (15.00–53.75)	22.50 (15.25–38.25)	0.388
SHBG (nmol/l)	56.90 (42.13–73.74)	67.58 (45.75–79.47)	0.053
Monocytes abs (Giga/l)	0.60 (0.50–0.70)	0.50 (0.40–0.60)	0.003
CRP (mg/l)	1.55 (0.76–3.67)	1.39 (0.73–2.51)	0.383
Troponin I (pg/ml)	8.05 (5.38–13.55)	5.40 (3.90–9.50)	<0.001
PTH 1–84 (pg/ml)	32.30 (27.25–47.33)	36.10 (29.30–46.20)	0.117
Vitamin D (ng/ml)	17.60 (14.00–21.33)	18.10 (14.00–21.60)	0.741
Calcium (mmol/l)	2.40 (2.30–2.50)	2.50 (2.40–2.50)	0.002
Urine Albumin (mg/l)	6.52 (3.65–18.65)	6.24 (3.40–13.98)	0.599
Urine Creatinine (mmol/l)	6.9 (5.07–9.4)	7.49 (4.4–10.40)	0.676

BMI: Body Mass Index. eGFR: estimated glomerular filtration rate. LDL: low density lipoproteins. HDL: high density lipoproteins. SCr: serum creatinine. SCys: serum Cystatin C. GGT: gamma-glutamyl transferase. SHBG: Sex hormone-binding globulin. CRP: C-reactive protein. PTH: Parathyroid hormone.Data shown are median (interquartile range, Q1–Q3).

*eGFR was calculated using serum Cystatin C and/or serum Creatinine following the equations described by Inker *et al*.^68^.

**p value was calculated using Mann Whitney U test (except for medication which was a Fisher’s exact test).

### Urine Metabolite Profiling of late-Onset Type 2 Diabetes Mellitus

**Figure 1:**
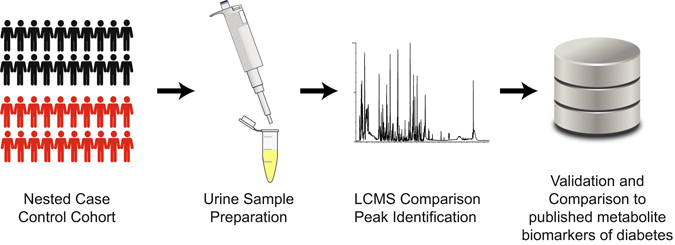
Workflow for untargeted metabolomics. Samples collected from nested case control cohort were prepared before being analyzed on UPLC-MS machines. After data preprocessing and data analysis, the metabolite biomarkers were validated using standards. The results are then compared to published biomarkers of diabetes.

**Figure 2:**
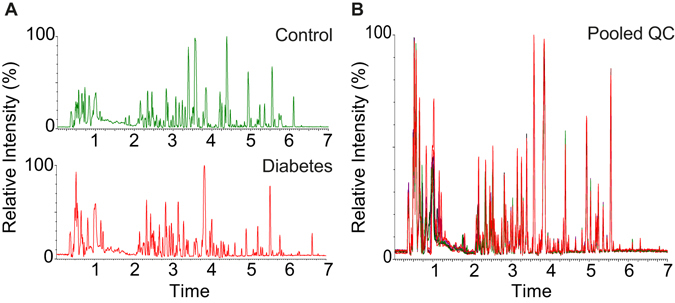
BPI chromatogram of urine samples. (**A**) Representative BPI chromatogram of urine samples from subjects with and without type 2 diabetes (T2D). (**B**) Overlap of QC samples BPI chromatogram shows that the retention time for major peaks were found to be stable throughout the whole analysis.

**Figure 3:**
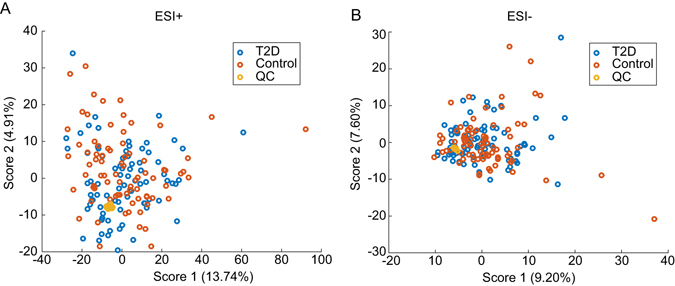
PCA was applied to features after preprocessing of LCMS measurements. The quality control (QC) samples were found to be well clustered near the center of the scores plot in both (**A**) positive and (**B**) negative ionization mode. Control and type 2 diabetes (T2D) samples, however, do not exhibit any meaningful separations. Quality of the dataset is first assessed using principle component analysis (PCA). Visual inspection of the clustering of the QC samples and drift of the run order QCs in the PCA scored plots were performed to assess the data integrity by tight clustering of the QC samples on the PCA score plots^63^. The percentage variance explained by each principle components is shown beside the axis legend.

An untargeted metabolomics workflow was applied in this study to characterize the metabolic profile and to identify metabolite biomarkers of older people with T2D (see Fig. 1). Representative base peak intensity (BPI) chromatograms of urine samples from older people with and without T2D are shown in Fig. 2A. By inspecting the Quality Control (QC) samples that were run in between the sample analysis, we note that retention time for major peaks were found to be stable without any discernable drifts in the peaks (see Fig. 2B). Next, peak alignment, peak picking, peak deconvolution, median normalization, and log transformation were applied on the raw UPLC-MS data. A total of 2,212 and 483 retention time-exact mass pairs (i.e. features) were found in each sample, in positive electrospray ionization mode (ESI+) and negative electrospray ionization mode (ESI−). The stability and the reproducibility of the sample analysis were visualized using Principal Component Analysis (PCA) score plots as shown in Fig. 3. A total of 19 QC samples were run throughout the entire analysis. The QC samples were found to be tightly clustered in the score plots, and there was no drift in their PCA scores. Majority of the features (99% in ESI+, and 98% in ESI−) were present in all the QC samples. After removing features that were not present in all the QC samples, the coefficient of variation (CV) or relative standard deviation (RSD) was calculated for all features. In the positive mode, 83% of the features’ CV was less than 10%, while 53% of features’ CV is less than 10% in the negative mode. Finally, the linearity of the features in the dilution QC samples was tested, 78% and 88% of features in positive and negative ionization were retained for downstream analysis when the threshold for acceptance was set at R^2^ > 0.9, and FDR adjusted *p* value < 0.10. After these steps, a total of 1,133 features were obtained from positive ionization, and 221 features from negative ionization.

To investigate if the variation in the measured metabolic profile can be attributed to the presence of type 2 diabetes mellitus, all observation acquired in both ionization modes, were first analyzed using two component PCA plots. We noted that the score plot did not show clustering of subjects with and without type 2 diabetes mellitus. Subsequently, a supervised approach, Orthogonal Projection to Latent Structure-Discriminant Analysis (OPLS-DA) modeling, was applied for further multivariate analysis.

### Multivariate Data Analysis of Urine Metabolic Profile

**Figure 4:**
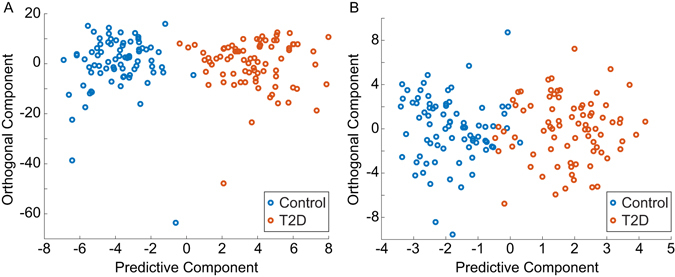
Multivariate data analysis of urine metabolomics data. (**A**) Scores plot of ESI+ measurements. The urine samples from diabetes subjects (T2D) and controls were found to be well separated along the predictive component axis with an explained variance (*R* ^*2*^ *Y*) of 0.84 and predictability (*Q* ^*2*^ *Y*) of 0.36. (**B**) Scores plot of ESI− experiment, with a *R* ^*2*^ *Y* of 0.77 and *Q* ^*2*^ *Y* of 0.46.

To obtain a list of features capable of defining the variation of the metabolic profiles of urine in subjects with and without type 2 diabetes, all observation acquired in both ionization modes were analyzed using OPLS-DA model^23, 24^. OPLS-DA modeling was performed with 5-fold cross-validation. Prior to the analysis, the data were standardized, and the model was adjusted for age, BMI, gender, and eGFR. In addition, the data was standardized for current metformin and statins medication using a dichotomous response value (yes/no). Different estimation of GFR using serum creatinine, serum cystatin C, or a combination of both was not found to affect the outcome of the results. The predictive and orthogonal score plots, adjusted for these confounders were found to discriminate older subjects with T2D from normoglycemic subjects (see Fig. 4). An explained variance (*R* ^*2*^ *Y*) of 0.84 and predictability (*Q* ^*2*^ *Y*) of 0.36 was obtained for ESI+. For ESI−, *R* ^*2*^ *Y* = 0.77 and *Q* ^*2*^ *Y* = 0.46. Label permutation^23^ was applied to validate the model and to confirm that the observed difference in metabolic profile did not arise from random variation (see Fig. S1). The metabolites were selected based on the absolute value of the model coefficients and the *p* value of the one way ANOVA after 10% false discovery rate (FDR) correction. In total, 17 metabolites and 2 metabolites from positive and negative ionization mode respectively, were selected for further metabolite structural assignments.

### Urine Biomarkers of T2D in Older People

**Figure 5:**
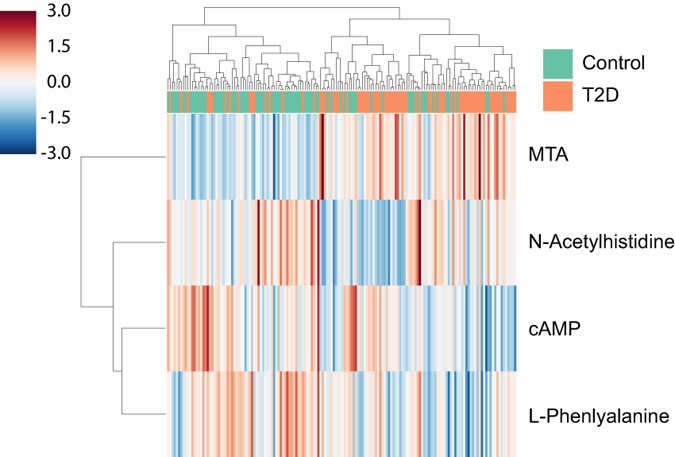
The relative abundance of biomarkers is shown as a heatmap. The color bar shows the abundance z-scores. Hierarchical clustering was applied to the validated biomarkers and cohort samples. The set of biomarkers are clustered into groups that are more or less abundant in the type 2 diabetes (T2D) group. The compounds at the top were found to be less abundant in T2D urine samples, while the compounds at the bottom are enriched in T2D urine samples.

Liquid chromatography tandem mass-spectrometry (LC-MS/MS) was performed on QC samples to gather fragmentation pattern of the 19 metabolite biomarkers selected from previously performed multivariate data analysis. The fragmentation patterns are matched against the theoretical fragmentation patterns of metabolites in HMDB. Metabolite candidates from HMDB were further validated by metabolite standards. Finally, we were able to validate the identity of 4 biomarkers using standards. The validated biomarkers in the urine samples match well with the retention time and *m/z* (see Table 2). In addition, the fragments *m/z* and their relative intensities were also found to be in absolute agreement with each other. The identity of the biomarkers, and their corresponding retention time, *m/z*, adduct, predictive component’s coefficient, ANOVA *p* values, FDR adjusted *p* values, and their fold change are listed in Table 3. Percentage fold change of specific biomarkers was calculated as the percentage change in the mean abundance of subjects with diabetes relative to the mean abundance of control subjects. Heat maps were constructed using the intensity value of the identified biomarkers, which show the relative levels of the four identified biomarkers are different between older subjects with and without chronic hyperglycemia (see Fig. 5). The intensity values indicated in the colorbar of Fig. 5 has been normalized using z-score. Urine samples from subjects with late-onset T2D were found to contain prominently elevated levels of 5′-methylthioadenosine (MTA) (*p* < 0.001), significantly lower levels of cAMP (*p* < 0.001), acetylhistidine (*p* < 0.001), and phenylalanine (*p* < 0.005). The abundance of MTA was found to be 34% higher in the urine of diabetic subjects, whilst urinary cAMP, acetylhistidine, and phenylalanine were found to be lower by 15%, 20%, and 12% respectively in the diabetic subjects.

**Table 2:** Comparison of retention time, precursor *m/z*, fragments *m/z*, and fragments relative intensity between biomarkers and standards.

Metabolite	Biomarker RT (min)/Precursor *m/z*	Standard RT (min)/Precursor *m/z*	MS Collision Energy (eV)	Biomarker *m/z* fragment, relative intensity	Standard *m/z* fragment, relative intensity
Cyclic AMP	2.24/330.0622	2.23/330.0622	20	136.0628, 100%	136.0652, 100%
				330.0622, 43%	330.0622, 90%
				312.0516, 6%	312.0516, 11%
Phenylalanine	2.50/166.0727	2.4/166.0885	10	166.0727, 100%	166.0885, 16%
				120.0820, 57%	120.0820, 100%
			20	120.0820, 100%	120.0820, 100%
				103.0548, 27%	103.0548, 21%
5′-methylthioadenosine (MTA)	3.16/298.0980	3.14/298.0980	10	298.0980, 100%	298.0980, 100%
				136.0628, 55%	136.0628, 50%
			40	136.0628, 100%	136.0628, 100%
				119.0356, 38%	119.0356, 27%
Acetylhistidine	0.54/198.0882	0.59/198.0911	10	198.0882, 100%	198.0911, 100%
				152.0818, 49%	152.0843, 37%
				156.0795, 34%	156.0795, 23%
				110.0727, 26%	110.0727, 15%
				180.0805, 20%	180.0805, 15%

**Table 3:** List of identified Biomarkers.

Compounds	Retention Time (mins)	*m/z*	Adduct	OPLS-DA Coefficient	ANOVA *p* value	FDR Corrected *p* value	Mean Fold Change (%)
cAMP	2.24	330.0602	M + H	−1.61E-2	3.16E-4	1.80E-2	−14.76
5′-Methylthioadenosine (MTA)	3.15	298.0973	M + H	1.72E-2	1.13E-4	7.56E-3	33.62
Acetylhistidine	0.58	198.0876	M + H	−1.85E-2	3.00E-5	2.85E-3	−19.84
Phenlyalanine	2.50	166.0838	M + H	−1.41E-2	1.75E-3	5.53E-2	−12.06

## Discussion

The majority of the water soluble waste products are removed by renal clearance. Therefore, urinary metabolites are enabling an insight into the body’s metabolic state, including various disease processes^25^. In our untargeted metabolite profiling study using LC/MS, we have uncovered evidence of perturbation in amino acid and nucleotide metabolites in subjects with late-onset type 2 diabetes mellitus. In diabetic subjects, the amino acids phenylalanine and acetylhistidine were found to be considerably decreased in comparison to controls. In addition, two novel biomarkers are identified in human urine, cAMP, and MTA, which until today, have only been implicated in animal models of diabetes^26, 27^. These biomarkers are further discussed below. Notably, in this study, substantial efforts have been expended towards the identification of biomarkers. In order for the identity to be accurately assigned, the urinary biomarkers and the procured standards are required to match all three orthogonal information, i.e. retention time, mass to charge ratio, and fragmentation pattern at different collision energy.

In our study, we found that phenylalanine was perturbed in diabetic subjects, consistent with previous metabolomics studies of “classical” T2D subjects^16, 19^. Even though phenylalanine has been widely reported as a biomarker of T2D in both urine and serum samples, previous studies have reported inconsistent results, either with elevated, unchanged, or reduced levels of this molecules^16, 19^. For example, using mass spectrometry analytical platform, phenylalanine has been reported to be lower in plasma samples of subjects with T2D^28^. In other studies, however, have found elevated phenylalanine in serum or plasma samples from subjects with T2D^29–31^, and prediabetes subjects^32, 33^. Studies using ^1^H NMR has similarly reported lower phenylalanine in serum of T2D subjects^34, 35^. In contrast to van Doorn and co-workers studying a cohort comprised of 11 T2D subject and 16 healthy subjects^36^, we found lower urinary phenylalanine in the late-onset subjects with T2D. The lack of consensus in the literature regarding urinary levels of phenylalanine could be at least partially attributed to dietary effects. Indeed, dietary lifestyle has been shown to affect the urinary metabolic profile^37, 38^. For example, the consumption of tea, coffee, wine, fruits, and vegetables has been associated with elevated levels of hippuric acids^39, 40^, while intake of chocolate and caffeic acid has been shown to result in elevated m-HPPA^41, 42^. Therefore, elevated levels of phenylalanine in some studies could potentially be attributed to the dietary use of non-nutritive sweeteners or food products that contain high amounts of phenylalanine and not the disease process per se. The decay of phenylalanine in the T2D subjects could be attributed to a greater usage of amino acids for gluconeogenesis due to compromised β-cells’ capacity to produce insulin as well as insulin action of insulin responsive tissues such as liver, and skeletal muscle^43^.

Histidine plays an important role in the regulation of methionine cycle and has been widely implicated in the chronic hyperglycemic state^16, 19^. Many studies have reported lower histidine level in both serum/plasma^44, 45^ and urine^46^ in diabetes subjects. Acetylhistidine, which is a derivative of histidine and a common urinary metabolite^47^ is also known to be elevated in the urine of patients with histidinaemia, where histidine levels are elevated in the blood and urine^48^. In our study, acetylhistidine was found to be significantly lower (*p* < 0.001) in the urine of older diabetes subjects. Interestingly, our finding in older adults is also in line with studies using animal models, where lower levels of acetylhistidine have been reported in the urothelial layer^49^ and urine^50^ of streptozotocin (STZ) induced diabetic rats.

Cyclic AMP (cAMP) is an important signaling metabolite in various pathways, and the primary effector of GLP-1/incretin induced insulin secretion from pancreatic β-cells^51^. cAMP and the associated PKA pathways are important in maintaining glucose homeostasis. The impairment of cAMP and PKA pathways in various organs, such as β-cells, α-cells, liver, skeletal muscle, adipose tissues, and the brain, have been implicated in the pathogenesis of T2D^52^. In this study, urinary cyclic AMP was found to be significantly lower in subjects with T2D (*p* < 0.001). Urinary cAMP has been reported to be considerably lower in an insulin-deficient rat model by Kodera *et al*.^26^. Other studies have found treatments with dipeptidyl peptidase-4 (DPP4) inhibitors resulted in a significant increase in urinary cAMP in T2D patients and rat models of insulin-deficiency suggesting a direct impact of the incretin system activity and urinary cAMP levels^50, 53^. To sum up these various findings, the fact that cAMP is perturbed in diabetes and can be modulated through diabetic treatments may suggest a possible systemic disturbance of cAMP/PKA pathways in older people with diabetes mellitus.

The sulfur-containing nucleoside 5′-methylthioadenosine (MTA), a key molecule in the purine salvage and methionine pathways^54^, was found to be higher in subjects with late-onset T2D in this study. A previous study has associated the elevated level of urinary MTA with severe combined immunodeficiency syndrome in humans^54^. While MTA is not well known to be connected to diabetes, recent evidence from a STZ (40 mg/kg) induced diabetic study in rats has indicated the possibility that MTA is elevated in the serum of diabetic rats^27^. In our study, we evidently indicate MTA as a possible biomarker based on the assignment, which was carefully performed on the procured standard using retention time, *m/z* and fragmentation pattern. Here, we are able to affirm that MTA is elevated in urine of older subjects with type 2 diabetes mellitus, which is consistent with a previous study in rat diabetes model^54^.

In this study, the classic antidiabetic drug metformin^55, 56^ was used in more than a third of the subjects with diabetes. In addition, almost half of the diabetic subjects were treated with statins, while 29.5% were medicated with metformin (see Table 1). This typical kind of polypharmacy in subjects with diabetes could potentially explain the lower cholesterol level observed in T2D subjects, but was not found to be significantly affected by metformin intake in T2Ds. Furthermore, metformin may exert a series of pleiotropic effects such the stimulation of ß-oxidation^57^ and may therefore alter lipids as well as amino acids in the serum^58–60^. Recently, metformin has also been shown to reduce citrulline levels in patients and murine tissues^61^. Urinary metabolites such as glutamic acid, methionine, panthothenic acid, and phenylalanine have also been reported to decrease in obese diabetes mouse model treated with metformin^57^. Therefore, in addition to the diabetes diet, the anti-diabetic medication applied could also modify the amount of urinary phenylalanine levels seen in subjects with T2D.

The major strengths of this study are unbiased metabolite profiling using ultra performance liquid chromatography – mass spectrometry (UPLC-MS) platform of human urine samples from a population-based nested case control cohort for the purpose of characterizing the metabolic profile and identification of urine biomarkers of T2D in older people. Mass spectrometry (MS) based approaches are highly sensitive and selective. MS also provides a wide coverage and allows high throughput analysis to be performed. However, several limitations have to be considered. The reverse phase column used in this study, do not provide a good coverage of hydrophilic compounds, because they are eluted at an early retention time, close to the solvent front. In addition, the sample size of the nested case control cohort used in this study is limited. There are also significant differences in the age and gender distribution amongst cases and controls, which, however, have been controlled in multivariate analysis. Due to the absence of older adults with earlier onset of T2D in this cohort, it is not possible to conclude if the biomarkers identified in this study are unique to older individuals with late-onset T2D. Therefore, a cohort study which includes older people with T2D onset at an earlier age can be included in future studies to identify biomarkers that are unique to late-onset and early onset T2D in older people.

## Conclusion

We applied untargeted metabolite profiling to urine samples in order to specifically address the metabolic profile of late-onset type 2 diabetes mellitus. Four novel metabolite biomarkers in the urine have been successfully identified, suggesting potential differences in the profile of older- versus medium-age onset type 2 diabetes mellitus. Two amino acids, namely phenylalanine and acetylhistidine, were found to be reduced in urine samples of late-onset T2D subjects. This is in agreement with the majority of the studies in younger-onset T2Ds that reported perturbations in urinary amino acids in the context of chronic hyperglycemia. Furthermore, the untargeted approach taken in this study has also identified two metabolites that have been implicated in animal models of diabetes mellitus. The sulfur-containing nucleoside MTA was found to be elevated in people with diabetes, while cyclic adenosine monophosphate (cAMP) was found to be lower. To the best of our knowledge, MTA and cAMP have not been reported as a validated biomarker of T2D in human urine.

## Methods

### Study Population

The ActiFE Ulm (Activity and Function in the Elderly in Ulm) study is a population-based cohort study in older people (>65 years), located in Ulm, State Baden-Wuerttemberg, and adjacent regions in Southern Germany. A detailed description of the cohort and the measures taken were previously described^20^. Briefly, a random sample of 7,624 non-institutionalized inhabitants was contacted by mail and invited to participate. Exclusion criteria were severe deficits in cognitive, vision, and hearing that precluded the accomplishment of most assessments or serious German language difficulties. Baseline assessments were completed by trained research assistants using standardized methods and included n = 1,506 eligible participants aged 65 or older. Inclusion criteria for the current metabolomic substudy were chronic hyperglycemia with an onset of the disease after age of 70 years or an absence of diabetes mellitus according to 2010 ADA criteria (see before); the study cohort comprised of 80 participants with diabetes and 78 controls, the latter were selected in a consecutive manner from the overall study participants without diabetes. Within this population based case-control study, HbA1c for older subjects with diabetes was 40.8 mmol/mol (IFCC; 5.9% DCCT/NGSP, translating into an average blood glucose level of 122 mg/dl). This value is lower compared to what has been reported HbA1c of 50.9 mmol/mol (IFCC; 6.8% DCCT/NGSP, translating into an average blood glucose level of 148 mg/dl) and 51.0 mmol/mol (IFCC; 6.8% DCCT/NGSP, translating into an average blood glucose level of 149 mg/dl) from the KORA and the HNR cohorts that have also included elderly people with T2D^62^. All participants provided written informed consent. The study was approved by the ethical committee of Ulm University, Ulm, Germany (IRB Application No. 318/08) and all methods were performed in accordance to the relevant guidelines and regulations.

### Sample Preparation

Urine samples from participants were collected in the morning after at least 12 hours of fasting conditions. The urine samples were collected during the visit and then immediately aliquoted and frozen at −80 °C until sample preparation for UPLC-MS analysis. For urine profiling study, the samples are prepared according to the protocols detailed by Want and co-workers^63^. Briefly, 60 μl of urine samples were centrifuged at 10,000 g for 10 minutes at 4 °C to remove particulates. Next, 50 μl of urine were mixed with 100 μl of UPLC-MS grade water. Finally, the samples were aliquoted appropriately and equal aliquots of each urine sample were pooled to form quality control (QC) sample. Since features that are not detected in the QC samples have been excluded from further analysis, metabolites that appear only in diabetes samples in low abundance will be diluted to below detection limit during the formation of pooled QC. Therefore, it is possible that biomarkers that appeared exclusively in the diabetes samples and in low abundance could not be detected in this study. Prior to data acquisition, samples were randomized and stored in the sample manager of Waters Acquity UPLC system with the temperature set at 4 °C.

### Urine Metabolite Profiling using UPLC-MS

Methods of urine metabolite profiling were adapted from a previously published protocol^63^, and performed on ACQUITY UPLC/Xevo G2-XS QTof (Waters, Manchester, UK) equipped with an electrospray source operating at either positive (ESI+) or negative ionization mode (ESI−). The source temperature was set at 120 °C with a cone gas flow of 50 L/h, a desolvation gas temperature of 450 °C with a desolvation gas flow of 1000 L/h. The capillary voltage set to 2 kV in both positive ionization mode, and 1.8 kV in negative ionization mode. The cone voltage was set to 30 V.

A 3 μl of sample was injected into 100 mm × 2.1 mm, 1.7 μm HSS T3 column (Waters) held at 40 °C using the ACQUITY UPLC system from Waters. Elution was performed with a linear gradient of 1–15% B over 1–3 minutes, 15–50% B over 3–6 minutes, 50–95% B over 6–9 minutes, and finally the gradient was held at 95% for 1.1 minutes. In both positive and negative ionization mode, mobile phase A is water with 0.1% formic acid and mobile phase B is acetonitrile containing 0.1% formic acid. The column flow rate was 0.5 ml/min.

Profile data were collected from 50 to 1,200 *m/z* for both positive and negative ionization mode with a scan time of 0.15 s over a 12 min analysis. Leucine enkephalin at a concentration of 200 ng/ml with a flow rate of 5 ml/min was used as the lock mass, which in positive ion mode has an *m/z* of 556.2771 and 554.2615 in negative ionization mode^64^. MassLynx software from Waters was used to control the system and data acquisition.

The UPLC-MS analysis in this study employed a QC strategy that was previously described by Gika *et al*.^65^. Firstly, to condition the column, QC sample was injected 12 times before initiating the run. Next, the QC sample was injected every 9 sample injection, and at the start and end of the analysis run. During the sample analysis, a total of 17 QC sample were injected, for the purpose of monitoring instrument stability and analyte reproducibility. After sample analysis, a series of diluted QC sample (1:9, 1:4, 1:2, 1:1) in the reconstitution solvent mixture was injected. Finally, a blank sample was injected at the end and start of the analysis.

### Data Preprocessing

Preprocessing of MS data (in RAW format), which includes automatically alignment using retention time, peak picking, and deconvolution, was performed using Progenesis QI v2.0 (Nonlinear Dynamics, Newcastle, UK). Samples were median normalized and log transformed^66^. Features near the solvent front, with a retention time less than 0.55 minute, and chromatographic peak width less than 0.03 minute were not included for further analysis. Features with intensity less than 3,000 were also discarded. A data matrix containing the samples analyzed versus detected features and their corresponding raw and normalized abundance values were produced for downstream analysis and processing in Python and MATLAB (Mathworks, Natick, MA). Using the QC samples, the unreliable features were removed following the procedures outlined in a previous publication^63^. Features were only accepted if they were present in all of the QC samples, and revealed a coefficient of variation (CV) less than 10%. Finally, raw abundance of features that did not display good linearity in the dilution QC samples, as defined by R^2^ < 0.9 and *p* value > 0.05, were also excluded from downstream analysis.

### Multivariate Data Analysis

Principle component analysis was performed in MATLAB and Python to visualize clustering and identify outliers. Orthogonal projection to latent structure (OPLS) analysis was performed to maximize separation between case and control samples while minimizing variability unrelated to the separation using the “ropls” package^67^ implemented in R. The measurement values were standardized prior to OPLS analysis and adjusted for age, BMI, sex, metformin medication, statins medication, and estimated glomerular filtration rate (eGFR). Estimation of eGFR was performed using serum creatinine, serum cystatin C, and a combination of both following the equations detailed in ref. 68 to assess the sensitivity of this estimation on the final results. Estimations were performed using three different equations, and their values are denoted as, eGFR–SCr, eGFR–SCys, and eGFR-SCr&SCys respectively (see Table 1). The optimal number of orthogonal components was determined using 5-fold cross validation. The *R* ^*2*^ *Y* was calculated to provide an indication of the variability explained by the model and the cross validated *Q* ^*2*^ *Y* was calculated to indicate the model performance in cross validation datasets^23^. For biological models, an acceptable criteria of Q^2^Y >  = 0.4 has been proposed^23^. The OPLS model was also further validated by label permutation^23^, where labels were resampled 999 times, generating a distribution of R^2^Y and Q^2^Y values when the models are fitted to non-informative data. The quality of the model was assessed by the R^2^Y and Q^2^Y statistics between the permuted and original label (see Fig. S1). After statistical modeling, top ranking features with FDR adjusted *p*-value < 0.1, were selected for further downstream identification.

### Metabolite Structural Assignment

To elucidate the structure of biomarkers, fragmentation pattern were obtained using UPLC-MS/MS at different collision energies, i.e. 10 eV, 20 eV, and 40 eV. Assignment of biomarker identity was performed by matching measured fragmentation pattern and accurate *m/z* of detected chromatographic peaks with the *m/z* and theoretical fragmentation pattern of compounds from the Human Metabolome Database (HMDB version 3.0)^69^ using CFM-ID^70^ and myCompoundID^71^. Spectra produced from collision energy that exhibit inadequate signal were not used for structural elucidation. Next, standard of metabolite was procured and run using identical UPLC-MS/MS condition. The measured retention time, *m/z*, and MS/MS spectrum of standard were matched to the biomarker obtained from standards. Standards were procured from Sigma-Aldrich, Singapore and AKos GMBH, Germany.

## Electronic supplementary material

Supplementary Information
