# Supplementary material for: Metabolite profiling in identifying metabolic biomarkers in older people with late-onset type 2 diabetes mellitus
Source: Sci Rep. 2017 Jun 29;7:4392. doi: 10.1038/s41598-017-01735-y (PMC5491522; doi:10.1038/s41598-017-01735-y)
Supplement: Supplementary file 1 — Supplementary Information [file 41598_2017_1735_MOESM1_ESM.pdf]

Metabolite profiling in identifying metabolic biomarkers in older people with late-onset type 2 diabetes mellitus

Zhi Yang Tam[1,2], Sean Pin Ng[1,2], Ling Qiao Tan[1,2], Chih-Hsien Lin[1,2], Dietrich Rothenbacher[3], Jochen Klenk[3,4], SPC Team, the ActiFE Study Group, Bernhard Otto Boehm[1,2,5,6]

1 Singapore Phenome Center, Experimental Medicine Building, Nanyang Technological University, 59 Nanyang Drive, Singapore 636921

2 Lee Kong Chian School of Medicine, Nanyang Technological University, 59 Nanyang Drive, Singapore 636921

3 Institute of Epidemiology and Medical Biometry, Ulm University, Helmholtzstrasse 22, 89081 Ulm, Germany

4 Department of Clinical Gerontology, Robert-Bosch-Hospital, Auerbachstrasse 110, 70376 Stuttgart, Germany

5 Imperial College London, London, UK

6 Department of Internal Medicine I, Ulm University Medical Centre, Ulm University, Albert-Einstein-Allee 23, 89081 Ulm, Germany

Correspondence to: Bernhard Boehm: bernhard.boehm@ntu.edu.sg

Supplementary Table

Table S1 Catalog numbers and CAS numbers of the standards used for structural assignment

No.	Biomarker	CAS Number	Catalogue Number
1.	Cyclic AMP	60-92-4	A9501 Sigma
2.	5'- methylthioadenosine (MTA)	2457-80-9	D5011 Sigma
3.	Phenylalanine	63-91-2	78019 Sigma
4.	Acetylhistidine	-	AKOS006238364

Supplementary Figure

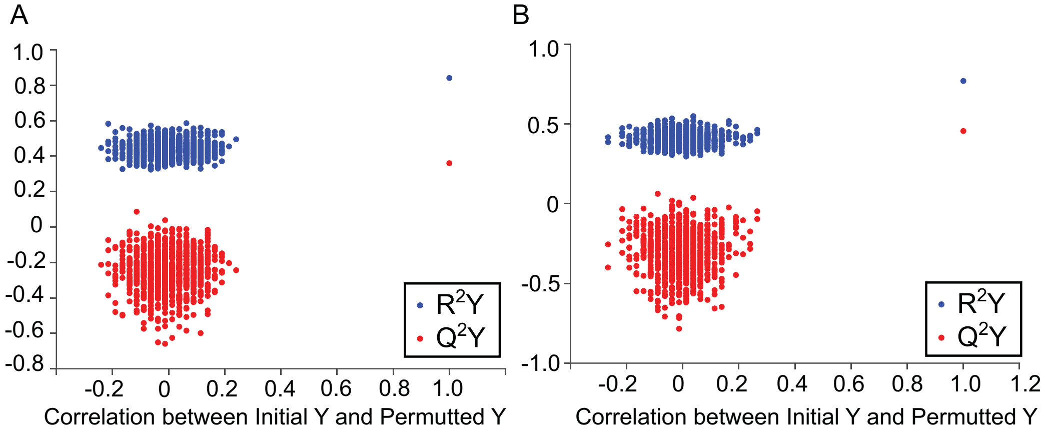

Figure S1 Result of model validation using label permutation for a) positive ionization data, and b) negative ionization data.
